# Dexamethasone affects cell growth/apoptosis/chemosensitivity of colon cancer via glucocorticoid receptor α/NF-κB

**DOI:** 10.18632/oncotarget.18802

**Published:** 2017-06-28

**Authors:** Jianming He, Jinming Zhou, Weiwen Yang, Qi Zhou, Xi Liang, Xueli Pang, Jianjun Li, Feng Pan, Houjie Liang

**Affiliations:** ^1^ Department of Oncology and Southwest Cancer Center, Southwest Hospital, Third Military Medical University, Chongqing 400038, China; ^2^ Department of Radiotherapy, Hebei Provincial Hospital of Traditional Chinese Medicine, Hebei University of Chinese Medicine, Shijiazhuang 050011, China; ^3^ Department of Radiology, Hebei Provincial Hospital of Traditional Chinese Medicine, Hebei University of Chinese Medicine, Shijiazhuang 050011, China

**Keywords:** glucocorticoid, glucocorticoid receptor, NF-κB, colon cancer, lymphoma

## Abstract

Glucocorticoids are effective to treat lymphoma and leukemia. Their effect in colon cancer remains far from clear. Here, we found that glucocorticoid receptor (GR) α protein level was dramatically lower in colon cancer than in lymphoma. Colon cell lines LoVo and HCT116 were GRα-rich and GRα was not detectable in HT29 or SW480. Dexamethasone significantly inhibited cell growth of GRα-rich cell lines and did not significantly affect GRα-negative cell lines. Dexamethasone induced apoptosis and increased chemosensitivity of GRα-rich cell lines. Knockdown of GRα significantly attenuated dexamethasone effects on cell growth, apoptosis and chemosensitivity. NF-κB p65 significantly correlated with GRα in colon cancer samples. Dexamethasone decreased NF-κB p65 activity. Knockdown of NF-κB p65 increased apoptosis. Our data demonstrate GRα protein level is dramatically lower in colon cancer than in lymphoma. Dexamethasone inhibits cell growth, induces apoptosis and enhances chemosensitivity in colon cancer, at least partly, via GRα and NF-κB.

## INTRODUCTION

Glucocorticoids have unique property to cause massive cell death and cell cycle arrest in malignant cells from the lymphoid lineage [1–2]. But they are generally not as effective in the treatment of non-hematological cancers [2–4]. Even so, glucocorticoids are commonly used in non-hematological tumors to relieve symptoms of cancers and treatments [2–4]. The use of glucocorticoids was associated with an increased response rate and a trend toward increased survival of patients with liver metastases from colorectal cancer (CRC) [4]. The mechanism is far from clear [2–4].

The main function of glucocorticoids is binding to their intracellular receptor, the glucocorticoid receptor (GR) [2, 5–6]. GR mainly exerts genomic or nongenomic effects in nucleus. It can also interact with cellular membranes or cytosolic targets to trigger certain signaling cascades [2, 5, 7–8]. The expression of GR, the functions of GR and glucocorticoids are virtually cell type-specific [2–3, 5–6, 9–10]. This study explored the effects and mechanism of dexamethasone (a type of glucocorticoids) in colon cancer and lymphoma.

## RESULTS

### Colon cancer expresses dramatic lower GRα than lymphoma and only partial colon cancer cells express detectable GRα

**Figure 1:**
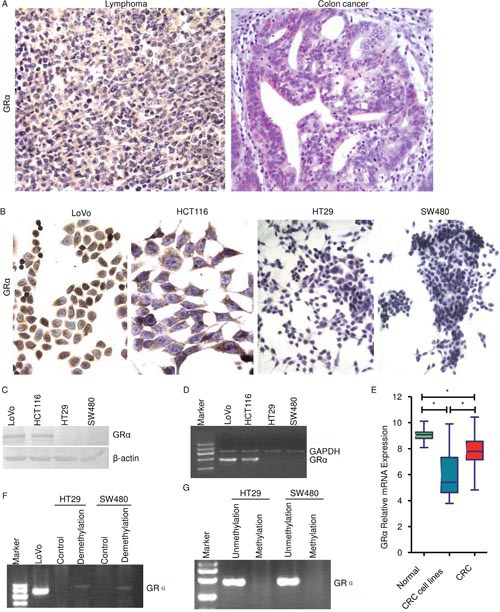
Colon cancer expresses dramatic lower GRα than lymphoma **(A)** GRαimmunohistochemistry of lymphoma and of colon cancer. **(B)** GRαimmunohistochemistry of colon cancer cell lines. **(C)** Western blot. **(D)** RT-PCR. **(E)** Microarray analysis showed that GRα mRNA level was significantly lower in CRC and in CRC cell lines than in normal colon epithelium. The microarray datasets were obtained from the publicly available NCBI Gene Expression Omnibus databank. **(F)** Cells were treated with or without the demethylating agent 5-Aza and GRα mRNA was assayed using RT-PCR. **(G)** Representative MSP-PCR results. A visible PCR product in lanes unmethylation indicates the presence of unmethylated alleles whereas a PCR product in lanes methylation indicates the presence of methylated alleles.

Immunohistochemical staining of GRα of archival surgical resection specimens from 10 patients with lymphoma (5 male; 5 female) and from 61 patients with colon cancer was analyzed (Figure 1A). GRα protein level was dramatically lower in colon cancer than in lymphoma. Almost all lymphoma cells were strong positive. Most of colon cancer cells were negative, a small part was weak positive and none was strong positive (Figure 1A). GRα in human CRC cell lines was evaluated using immunohistochemistry, western blot and semi-quantitative reverse transcription polymerase chain reaction (RT-PCR), respectively (Figure 1B–1D). Immunohistochemistry showed that GRα was positive in LoVo and HCT116. GRα was not detectable in HT29 or SW480 (Figure 1B). Western blot and RT-PCR showed that GRα was expressed in both LoVo and HCT116 while it was not detectable in HT29 or SW480 (Figure 1C and 1D). Microarray analysis showed that GRα mRNA level was significantly lower in CRC and in CRC cell lines than in normal colon epithelium (Figure 1E). These prove that GRα protein level is low in CRC and only partial CRC cells express detectable GRα.

In some cell types, the dominant mechanism of GR silencing is through methylation [2]. HT29 or SW480 cells were treated with the demethylating agent, 5-Aza-2’-deoxycytidin (5-Aza) [11–12]. Result of RT-PCR showed that 5-Aza treatment did not significantly increased GRα mRNA (Figure 1F). The promoter methylation status of GR was analyzed by methylation-specific PCR (MSP-PCR), a method allowing for distinction between unmethylated and methylated alleles [11–12]. MSP-PCR analyses verified that HT29 or SW480 displayed an unmethylated allele and GR methylated allele was not detectable (Figure 1G). As mentioned above, GRα was not detectable in HT29 or SW480 (Figure 1B–1D). Taken together, these imply that low expression of GRα in colon cancer might not be due to methylation.

### GRα correlates with colon cancer differentiation

Archival surgical resection specimens from 61 patients with colon cancer with a mean age of 55 (range 21–84) years were analyzed. Of the 61 cancer samples, 28 were in the right colon and 33 were in the left. 12 were Dukes’ stage A, 26 were stage B, 18 were stage C and 5 were stage D. 7 were well differentiated, 41 were moderately differentiated and 13 were poorly differentiated. All samples underwent routine immunohistochemical staining for GRα protein.

Clinical and pathological features of patients and their correlation with the presence of immunohistochemical staining of GRα are summarized in Table 1. Positive GRα expression was found in 35 of 61 (57%) cases of colon cancer examined, while it was negative in the remaining 26 (43%). Of the 35 GRα positive cases, 31 (88.6%) were well to moderate differentiated while 4 (11.4%) were poor differentiated. Of the 26 GRα negative cases, 17 (65.4%) were well to moderate differentiated while 4 (34.6%) were poor differentiated. The presence of positive immunohistochemical staining for GRα significantly correlated with cancer differentiation (P=0.029). No statistically significant difference in GRα expression was found concerning sex, age, Dukes’ stage, or lymph node metastasis.

**Table 1:** Spearman's rank correlation coefficient analysis of the association between presence of immunohistochemical staining of GRα and NF-κB, and clinicopathological parameters in patients with colon cancer (n=61)

Parameter	GRα postive n(%)	GRα negative n(%)	*P*
Sex			0.355
Male, n=30	19(63.3)	11(37.7)	
Female, n=31	16(51.6)	15(48.4)	
Age, years			0.973
<60, n=28	16(57.1)	12(42.9)	
≥60, n=33	19(57.6)	14(42.4)	
Cancer differentiation grade			0.029
Well to Moderate, n=48	31(64.6)	17(35.4)	
Poor, n=13	4(30.8)	9(69.2)	
Dukes’ stage			0.916
A and B, n=38	22(57.9)	16(42.1)	
C and D, n=23	13(56.5)	10(43.5)	
Lymph node metastasis			0.241
Positive, n=19	13(68.4)	6(31.6)	
Negative, n=42	22(52.4)	20(47.6)	
NF-κB			0.005
Positive, n=40	28(70.0)	12(30.0)	
Negative, n=21	7(33.3)	14(66.7)	

### GRα agonist, dexamethasone, inhibits cell growth, induces apoptosis and enhances chemosensitivity in colon cancer

The above data demonstrate that though GRα expression is low in colon cancer *in vivo* on the whole, there are still some colon cancer cells expressing detectable GRα. It was unclear whether glucocorticoids affected GR-rich CRC cells [4, 9]. Correlation between GRα and cell cycle genes/apoptosis genes in CRC was analyzed. The heatmap showed that GRα strongly correlated with some cell cycle genes/apoptosis genes (Supplementary Figure 1).

**Figure 2:**
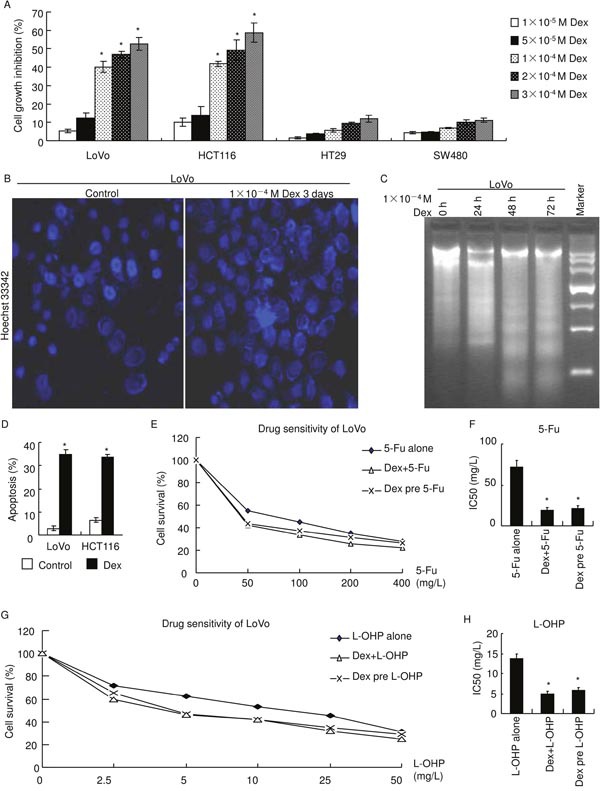
GRα agonist, dexamethasone, inhibits cell growth, induces apoptosis and enhances chemosensitivity **(A)** Cells were treated with dexamethasone as indicated for 3 days. The absorbance was measured using MTT assay and normalized to their control cells (without dexamethasone), respectively.(*:*p*<0.05 *vs* 1×10^−5^ M) **(B)** Nucleus was stained with Hoechst 33342. Dexamethasone increased apoptosis. **(C)** DNA fragmentation. Dexamethasone increased apoptotic DNA fragmentation. **(D)** Cells were treated with 1×10^−4^ M dexamethasone for 72 hours and apoptosis was measured using flow cytometry after FITC Annexin V/PI staining. (*:*p*<0.05 *vs* control) **(E, F)** LoVo cells were treated with 5-Fluorouracil and cell survival was assayed using MTT. IC50 was calculated due to Fig. 2E. (*:*p*<0.05 *vs* 5-Fu alone) **(G, H)** LoVo cells were treated with oxaliplatin and cell survival was assayed using MTT. IC50 was calculated due to Fig. 2G. (*:*p*<0.05 *vs* L-OHP alone).

To evaluate effect of glucocorticoids-GRα on cell growth in colon cancer, GRα-rich CRC cell lines (LoVo and HCT116) and GRα-negative CRC cell lines (HT29 and SW480) were treated with gradient dexamethasone for 3 days. Cell growth was assayed using MTT. Dexamethasone significantly inhibited cell growth of GRα-rich cell lines (LoVo and HCT116) in a dose dependent manner while it did not significantly change cell growth of GRα-negative cell lines (HT29 or SW480) (Figure 2A). In LoVo, cell growth inhibition ratios of 1×10^−4^, 2×10^−4^ and 3×10^−4^ mol/L (M) dexamethasone were 40.2%, 46.9%, 52.6%, respectively. In HCT116, they were 41.8%, 49.3%, 58.8%, respectively.

Effect of dexamethasone on apoptosis was explored. First, LoVo nucleus was stained with Hoechst 33342 (Figure 2B). Living cells show dispersion, uniform fluorescence in nuclei [13]. Apoptotic cells show nuclear condensation or nuclear fragmentation with fluorescence in nucleus or cytoplasm [13]. Dexamethasone treatment increased apoptosis in LoVo (Figure 2B). Second, DNA fragmentation, a biochemical hallmark of apoptosis, of LoVo cells incubated with 1×10^−4^ M dexamethasone for 24, 48 or 72 hours were separated using 1.8% gel electrophoresis (Figure 2C). Results verified dexamethasone increased apoptotic DNA fragmentation. Last, apoptosis was measured using flow cytometry after FITC Annexin V/propidium iodide (PI) staining (Figure 2D). LoVo and HCT116 were incubated with or without 1×10^−4^ M dexamethasone for 72 hours. Apoptosis ratio of LoVo treated with dexamethasone was 34.8±1.9% and that of control was 2.9±0.4% (*p*<0.01). Apoptosis ratio of HCT116 treated with dexamethasone was 33.6±1.4% and that of control was 6.4±1.3% (*p*<0.01). Collectively, these demonstrate that GRα agonist, dexamethasone, induces apoptosis.

Cell growth and apoptosis play key roles in chemosensitivity [14–16]. So, MTT assay was applied to evaluate effect of dexamethasone on chemosensitivity (Figure 2E–2H). LoVo was treated with 5-Fluorouracil (5-Fu alone), oxaliplatin (L-OHP alone), 5-Fluorouracil combined with 1×10^−4^ M dexamethasone (Dex+5-Fu), oxaliplatin combined with 1×10^−4^ M dexamethasone (Dex+L-OHP), 5-Fluorouracil following 1×10^−4^ M dexamethasone (Dex pre 5-Fu) 24 hours treatment, or oxaliplatin following 1×10^−4^ M dexamethasone 24 hours treatment (Dex pre L-OHP). Cells were incubated with anticancer drugs for 24 hours. Results showed that dexamethasone, regardless combined with or prior to anticancer drugs, significantly enhanced chemosensitivity (Figure 2E–2H). The half maximal inhibitory concentration (IC_50_) of 5-Fluorouracil were 72.2±8.1 mg/L in 5-Fu alone, 18.6±4.0 mg/L in Dex+5-Fu and 21.1±4.1 in Dex pre 5-Fu (Figure 2F). IC_50_ of oxaliplatin were 13.7±1.3 mg/L in L-OHP alone, 4.8±0.7 mg/L in Dex+L-OHP and 5.9±0.6 mg/L in Dex pre L-OHP (Figure 2H).

### Dexamethasone functions, at least partly, through GRα in colon cancer

**Figure 3:**
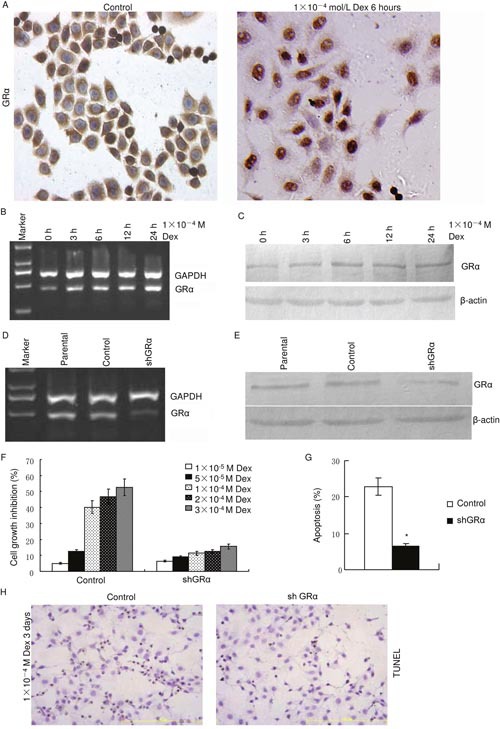
GR agonist, dexamethasone, functions through GRα in LoVo **(A)** Cells were treated with dexamethasone as indicated and GRα was stained usingimmunohistochemistry. **(B)** Cells were treated with dexamethasone as indicated and GRα mRNA was evaluated using RT-PCR. **(C)** Cells were treated with dexamethasone as indicated and GRα protein was evaluated using western blot. **(D)** RT-PCR. GRα mRNA was knocked down using shRNA targeting GRα (shGRα). **(E)** Western blot. GRα protein was decreased using shRNA. **(F)** Dexamethasone significantly inhibited cell growth of LoVo control cells and knockdown of GRα reduced the cell growth inhibition of dexamethasone. There was a significant difference between control group and shGRα group (*p*<0.05). **(G)** Cells were treated with 1×10^−4^ M dexamethasone for 72 hours and apoptosis was measured using flow cytometry after FITC Annexin V/PI staining. (*:*p*<0.05 *vs* control) **(H)** LoVo cells were treated with dexamethasone as indicated and apoptotic cells were stained using TUNEL.

Immunohistochemistry showed that GRα resides predominantly in the cytoplasm of control LoVo cells (Figure 3A). GRα mainly traveled to the nucleus 6 hours after 1×10^−4^ M dexamethasone treatment (Figure 3A). RT-PCR and western blot showed that 1×10^−4^ M dexamethasone increased GRα mRNA and protein in a time dependent manner (Figure 3B and 3C).

To explore the role of GRα in dexamethasone functions in LoVo cells, GRα was knocked down by small hairpin RNA (shRNA). RT-PCR and western blot confirmed knockdown efficiency (Figure 3D and 3E). Cell growth inhibition was evaluated by MTT assay (Figure 3F). Dexamethasone significantly inhibited cell growth of LoVo control cells in a dose dependent manner (Figure 3F), similar as in LoVo parental cells (Figure 2A). Knockdown of GRα reduced the cell growth inhibition of dexamethasone. There was a significant difference between control cells and shGRα cells (*p*<0.05). These indicate that dexamethasone inhibits cell growth via GRα. LoVo control cells and shGRα cells were incubated with 1×10^−4^ M dexamethasone for 72 hours and apoptosis was measured using flow cytometry after FITC Annexin V/PI staining (Figure 3G) and using TUNEL (Figure 3H), respectively. Flow cytometry showed that apoptosis ratio was significantly lower in shGRα cells than in control cells (*p*<0.01) (Figure 3G). Result of TUNEL was consistence with Flow cytometry. Dexamethasone treatment induced less apoptosis in shGRα cells than in control cells (Figure 3H). Taken together, these prove that dexamethasone inhibits cell growth and induces apoptosis, at least partly, through GRα.

### GRβ decreases apoptosis induced by dexamethasone in colon cancer

**Figure 4:**
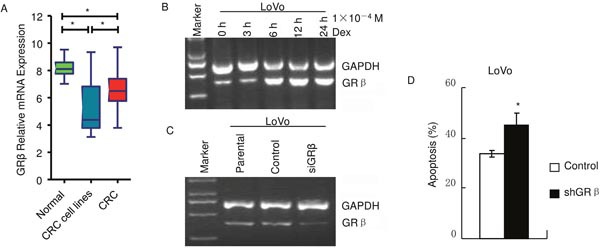
GRβ decreases apoptosis induced by dexamethasone in LoVo **(A)** Microarray datasets showed CRC or CRC cell lines had a significant lower GRβ mRNA level than Normal samples. (*:*p*<0.01) **(B** and **C)** RT-PCR. **(D)** Cells were treated with 1×10^−4^ M dexamethasone for 72 hours and apoptosis was measured using flow cytometry after FITC Annexin V/PI staining. (*:*p*<0.05 *vs* control).

GRβ is an isoform of GRα and it has a shortened ligand-binding domain that cannot bind glucocorticoids [2, 6, 9]. When coexpressed with GRα, GRβ can function as a dominant negative splice variant of GRα [2, 6, 9]. Microarray analysis showed that GRβ protein level was significantly lower in CRC and in CRC cell lines than in normal colon epithelium (Figure 4A), similar as GRα (Figure 1E). RT-PCR showed that LoVo cells also expressed GRβ and 1×10^−4^ M dexamethasone increased GRβ mRNA level in a time dependent manner (Figure 4B). GRβ was knocked down by the small interfering RNA (siRNA) sequences (Figure 4C). Apoptosis was measured using flow cytometry after FITC Annexin V/PI staining (Figure 4D). LoVo control cells and siGRβ cells were incubated with 1×10^−4^ M dexamethasone for 72 hours. Knockdown of GRβ increased apoptosis induced by dexamethasone. Apoptosis ratio of siGRβ cells was 45.4±4.3% and that of control was 33.8±1.5% (*p*<0.05). These suggest that GRβ decreases apoptosis induced by dexamethasone.

### Dexamethasone suppresses NF-κB activity in colon cancer

**Figure 5:**
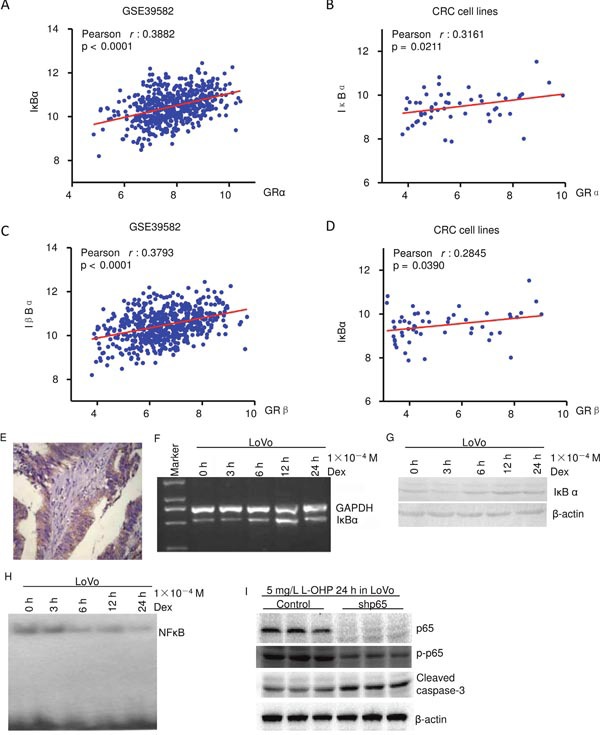
Dexamethasone suppresses NF-κB activity in colon cancer **(A–D)** Microarray datasets from CRC patients (GSE39582) and from CRC cell lines showed IκBα significantly correlated with GRα **(A** and **B)**, and with GRβ **(C** and **D)**. **(E)** NF-κB p65immunohistochemistry of colon cancer. **(F)** RT-PCR. **(G)** Western blot. **(H)** LoVo cells were treated with dexamethasone as indicated and NF-κB activity was assayed using EMSA. **(I)** Western blot. Knockdown of NF-κB p65 increased oxaliplatin induced cleaved caspase 3, an apoptotic marker.

NF-κB pathway was reported to play a key role of in apoptosis [2, 10, 17]. The heatmap showed that GRα strongly correlated with some apoptosis genes (Supplementary Figure 1). Further microarray analysis showed that both GRα and GRβ strongly correlated with IκBα, an inhibitor of NF-κB [10, 17], in CRC patients (GSE39582) and CRC cell lines (Figure 5A–5D). Accordingly, the role of NF-κB in dexamethasone inducing apoptosis in LoVo was explored. The correlation of GRα and NF-κB in human colon cancer was explored. NF-κB p65 protein level in human colon carcinoma samples was evaluated using immunohistochemistry (Figure 5E). Clinical and pathological features of patients and their correlation with the presence of immunohistochemical staining of NF-κB are summarized in Table 1. NF-κB p65 significantly correlated with GRα (P=0.005).

RT-PCR and western blot showed that 1×10^−4^ M dexamethasone increased IκBα in a time dependent manner (Figure 5F and 5G). Electrophoretic mobility shift assay (EMSA) showed that 1×10^−4^ M dexamethasone dereased NF-κB activity in a time dependent manner (Figure 5H). NF-κB p65 was knocked down by retroviral mediated shRNA interference [14, 18] (Figure 5I). NF-κB p65 knockdown cells (shp65) and control cells were treated with 5 mg/L oxaliplatin for 24 hours. Western blot showed that p65 and p-p65 was decreased by retroviral delivery of shRNA. Knockdown of NF-κB p65 increased oxaliplatin induced cleaved caspase 3, an apoptotic marker [15–16] (Figure 5I). These indicate that dexamethasone suppresses NF-κB pathway.

### Dexamethasone increases GR and suppresses NF-κB activity in lymphoma

Glucocorticoids remain one of the most effective and most commonly used drugs to kill lymphoma cells. The role and mechanism of glucocorticoids-GR in lymphoma has been well studied [1–2, 6, 8]. It seemed that colon cancer cell line LoVo and lymphoma cell line Jurkat shared the mechanism, glucocorticoids regulating GR to suppress NF-κB activity [2, 6]. Accordingly, effect of dexamethasone on GRα, GRβ, IκBα and NF-κB in the human T-lymphocyte cell line Jurkat was explored.

**Figure 6:**
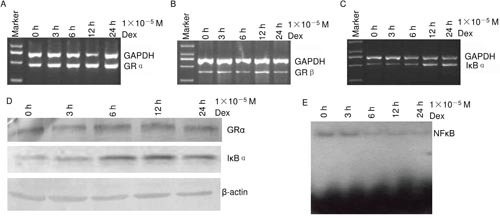
The effects of dexamethasone in Jurkat **(A–C)** RT-PCR. **(D)** Western blot. **(E)** NF-κB activity was assayed using EMSA.

Jurkat was treated with 1×10^−5^ M dexamethasone for different times. RT-PCR showed that dexamethasone increased GRα mRNA, GRβ mRNA and IκBα mRNA in a time dependent manner (Figure 6A–6C). Western blot showed that dexamethasone increased GRα protein and IκBα protein in a time dependent manner (Figure 6D). EMSA showed that dexamethasone decreased NF-κB activity in a time dependent manner (Figure 6E). These, together with other reports [2, 8], suggest that the human T-lymphocyte cell line Jurkat and the human colon cancer cell line LoVo may have a common mechanism, glucocorticoids suppressing NF-κB activity via GR.

The heatmap showed that GRα correlated with bcl-2 (Supplementary Figure 1). Results of RT-PCR and western blot showed that 1×10^−4^ M dexamethasone did not detectably change bcl-2 mRNA level or protein level in LoVo (Supplementary Figure 2A and 2B). Nor 1×10^−5^ M dexamethasone detectably changed bcl-2 mRNA level or protein level in Jurkat (Supplementary Figure 2C and 2D). The correlation of GRα and bcl-2 in human colon cancer was explored. Protein of bcl-2 of archival surgical resection specimens from 61 patients with colon cancer were stained with immunohistochemistry (Supplementary Figure 2E). The presence of positive immunohistochemical staining for bcl-2 did not significantly correlate with GRα (P=0.643) (Supplementary Figure 2F). These indicate that bcl-2 might not play a dominant role in the mechanism of glucocorticoids function.

## DISCUSSION

CRC is the third most commonly diagnosed cancer among men and women worldwide [19–20]. Chemotherapy remains one of principle ways to cure CRC [14–16]. Co-administration of glucocorticoids with chemotherapy is a common clinical practice used to prevent drug-induced allergic reaction or nausea/vomiting [2–4]. This study demonstrates that dexamethasone inhibits cell growth, induces apoptosis and enhances chemosensitivity of GRα-rich colon cancer cell lines (Figures 2 and 3). These suggest that glucocorticoids might be a potential solution to increase chemosensitivity of GRα-rich colon cancer or a potential target to treat it in clinic.

The principle function of glucocorticoids is via binding to GR. GRα is the predominant and functionally active receptor [2]. GRα has tissue-specific expression patterns that are conserved in different species [2]. GRα expression level was dramatically lower in colon cancer than in lymphoma, even lower than in normal colon epithelium (Figure 1A and 1E). GRα protein level in most of colorectal cancer cells was too low to be detected (Figure 1A–1C). These contribute to explain the fact that glucocorticoids are effective to treat lymphoma and leukemia while they are generally not as effective to treat CRC [2, 4, 8].

GRα is a ligand-inducible transcription factor and is mainly activated through binding to glucocorticoids [2, 5–6, 9–10]. In the absence of ligand, GRα resides predominantly in the cytoplasm. Upon ligand binding, the GRα complex changes its conformation and translocates to the nucleus [2, 5–6, 9–10]. This was also observed in GRα positive colon cancer cell LoVo. After treatment of dexamethasone, GRα mainly traveled to the nucleus (Figure 3A). Interestingly, dexamethasone increased GRα mRNA and protein level in a time dependent manner, through an unknown mechanism (Figure 3B and 3C).

Glucocorticoids functions as agonist of GRα through binding to GRα C-terminus containing important parts of the ligand binding domain [2, 5]. GRβ is unable to bind glucocorticoids because GRβ lacks the ligand binding domain [2, 5]. It is reported that GRβ has the capacity to dominant-negative inhibit GRα, either through competition for co-regulators or through formation of inactive GRα/GRβ heterodimers [2, 5]. In this study, dexamethasone increased GRβ mRNA level (Figure 4B). Knockdown of GRβ enhanced dexamethasone induction of apoptosis (Figure 4C and 4D). These provide evidences to support that GRβ may be also a dominant-negative inhibitor of GRα in colon cancer.

NF-κB plays a key role of in apoptosis. Suppression of NF-κB activation is a novel and common downstream of glucocorticoids activating GR in lymphoma, lymphoblastic leukaemia cells [2, 8]. IκBα, an inbitor of NF-κB [10, 17], strongly correlated with GRα and GRβ (Figure 5A–5D). Dexamethasone increased IκBα and suppressed NF-κB activity in colon cancer cells LoVo and the human T-lymphocyte cell line Jurkat (Figure 5F–5H, Figure 6C–6E). Knockdown of NF-κB p65 increased apoptosis (Figure 5I). These *in vitro* experiments indicate that suppression of NF-κB activation is a novel and common downstream of glucocorticoids, might through GR. *In vivo* experiment also showed that NF-κB expression significantly correlated with GRα (Figures 1A and 5E and Table 1). This supports the above conclusion based on *in vitro* experiments.

The correlation between GRα expression and clinicopathological parameters were also studies. Though GRα expression did not significantly correlate with cancer stage, lymph node metastasis, patient's sex or age, it significantly correlated with colon cancer differentiation (Figure 1A and Table 1). Taken together, these provide evidences supporting that GRα should play a role in colon cancer. In some cell types, the dominant mechanism of GR silencing is through methylation [2]. But it seems that this might not play a dominant role in GRα-negative colon cancer cell lines, HT29 or SW480 (Figure 1C–1G). The mechanism is far from clear.

In summary, GRα significantly correlated with colon cancer differentiation. Though colon cancer expressed dramatic lower GRα than lymphoma *in vivo*, part of colon cancer cells did express GRα. Dexamethasone inhibited cell growth, induced apoptosis and enhanced chemosensitivity in GRα-rich colon cancer cells via GRα. Suppression of NF-κB activation might be a novel and common downstream of glucocorticoids in colon cancer and lymphoma, likely via GR. These contribute to explain the difference in glucocorticoids effect in colon cancer and in lymphoma, and suggest that glucocorticoids-GR might be a potential solution to treat colon cancer in clinic. The detailed function and mechanism deserve further investigation.

## MATERIALS AND METHODS

### Patients and cancer samples

The archival colon cancer samples were derived from 61 patients undergoing surgical excision and diagnosed at the Department of Pathology, Southwest Hospital, Third Military Medical University. Patients were selected only based on acquirable tissues between 2003 and 2005. The patients who received radiotherapy or chemotherapy prior to surgery were excluded. All available hematoxylin and eosin (H&E) stained slides were reclassified by a pathologist without knowledge of patient's data. Tumor differentiation was graded as follows: well, moderate, or poor.

The archival lymphoma samples were derived from 10 patients undergoing surgical biopsy, diagnosed at the Department of Pathology, and re-diagnosed at the Department of Hematology, Southwest Hospital. Patients were selected only based on acquirable tissues between 2005 and 2006. The patients who received chemotherapy prior to surgery were excluded.

Informed consent was obtained from all patient involved in this study. This study was approved by the Ethics Committee of Southwest Hospital, the Third Military Medical University and was carried out in accordance with the associated guidelines.

### Cell lines and cell culture

Human colon cancer cell lines, LoVo, HCT116, HT29, SW480 and the human T-lymphocyte cell line Jurkat were obtained from the Cell Bank, Chinese Academy of Science. Cells were routinely grown and passaged as previously described [16, 21]. In brief, cells were grown in F12K (Gibco, Grand Island, NY, USA) (LoVo), McCoy's 5A (Gibco) (HCT116 and HT29), Leibovitz's L-15 (Gibco) (SW480), or RPMI-1640 (Gibco) (Jurkat) supplemented with 100 mL/L fetal bovine serum (FBS) (Gibco), 100,000 IU/L penicillin, and 100 mg/L streptomycin (Gibco) under a humidified atmosphere of 5% CO_2_ at 37°C.

### Treatment of 5-Aza

Cells were sequentially incubated with 2 mg/L 5-Aza (Sigma-Aldrich, St Louis, MO, USA)/media, fresh media, 4 mg/L 5-Aza/media, fresh media, 4 mg/L 5-Aza/media, fresh media for 24 hours. Then, cells were acquired [12].

### Treatment of dexamethasone

Cells were incubated with gradient concentrations of dexamethasone (Sigma-Aldrich)/media without FBS for different times as indicated. The control cells were incubated with vehicle (DMSO) in media without FBS.

### Immunohistochemistry

Immunohistochemistry of human samples was performed as previously described [12, 18]. In brief, 10% neutral formalin-fixed, paraffin-embedded tissues were cut into 5 to 6 μm thickness sections. Immunohistochemistry was done according to the manual of SP-9000-D kits (Streptavidin-Biotin) (Beijing Zhongshan Jinqiao biotechnology Co., Ltd., China). The applied primary antibodies consisted of the following: GRα (Santa Cruz Biotechnology, Santa Cruz, CA, USA), NF-κB p65 and Bcl-2 (Beijing Zhongshan Jinqiao biotechnology Co., Ltd.). Expression of GRα, NF-κB p65 or Bcl-2 was evaluated based on the percentage of positive cells. The slide with lower percentage of positive cells than or equal to 5% was considered as negative. The slide with higher percentage was considered as positive.

Immunohistochemistry of cultured cells was performed as previously described [15]. Cells were cultured on cover slides for 2 days. After fixation in 4% paraformaldehyde for 30 minutes, cells were stained using SP-9000-D kits (Beijing Zhongshan Jinqiao biotechnology Co., Ltd.) according to the manual.

### RT-PCR

RT-PCT was performed as previously described [18, 22]. In brief, total RNA of cells was extracted with Trizol reagent (Invitrogen, Carlsbad, CA, USA), and first-strand cDNA was synthesized using 2μg of total RNA. Then 1μL cDNA was taken as a template for the PCR reaction. GAPDH was amplified by PCR using the following cycle: initial denaturation at 94°C for 5 minutes, denaturation at 94°C for 45 sec, reassociation at 57°C for 45 sec, primer extension at 72°C for 45 sec (30 cycles), and a final extension step at 72°C for 10 minutes in the presence of Taq polymerase (TaKaRa Biotechnology Co. Ltd., Japan). GRα was amplified by PCR using the following cycle: initial denaturation at 95°C for 1 minutes, denaturation at 95°C for 30 sec, reassociation at 62°C for 30 sec, primer extension at 72°C for 30 sec (45 cycles). GRβ was amplified by PCR using the following cycle: initial denaturation at 94°C for 5 minutes, denaturation at 94°C for 45 sec, reassociation at 62°C for 45 sec, primer extension at 72°C for 60 sec (35 cycles). IκB was amplified by PCR using the following cycle: initial denaturation at 94°C for 5 minutes, denaturation at 94°C for 60 sec, reassociation at 55°C for 45 sec, primer extension at 72°C for 45 sec (35 cycles). Bcl-2 was amplified by PCR using the following cycle: initial denaturation at 94°C for 5 minutes, denaturation at 94°C for 60 sec, reassociation at 61°C for 45 sec, primer extension at 72°C for 45 sec (35 cycles). The PCR products were subjected to agarose gel electrophoresis with ethidium bromide. The primers used for RT-PCR are listed in Table 2. GAPDH was used as an internal standard.

**Table 2:** Primers for RT-PCR

GAPDH	Forward	CAAATTCCATGGCACCGTCA
	Reverse	GGAGTGGGTGTCGCTGTTGA
GRα	Forward	CCTAAGGACGGTCTGAAGAGC
	Reverse	GCCAAGTCTTGGGCCCTCTAT
GRβ	Forward	TACAAGCAGAACTGAGGCACT
	Reverse	CCTACAGCTACAGTCAGGGAGT
IκB	Forward	GCCTGCCACTCAGTGTATTT
	Reverse	GAGCCATCATCCGTTCTACC
Bcl-2	Forward	AGCCCAGACAAATGTGGTTAC
	Reverse	CCCCAATACAGGTCCTTCATA
M-GR	Forward	GGGACGGATTTTGTGGGTG
	Reverse	CCCCAATCCCCGAAACTAATA
U-GR	Forward	GGGATGGATTTTGTGGGTG
	Reverse	CCCCAATCCCCAAAACTAATA

### MSP-PCR

DNA was extracted using phenol-chloroform method as described [23]. DNA was modified by treatment with sodium bisulfite using EZ DNA Methylation kit (Zymo Research, Irvin, CA, USA) following the manufacturer's protocol [12]. MSP-PCR was performed using the M-GR and U-GR primer (Table 1) for methylated and unmethylated DNA, respectively. Each gene was amplified by PCR using the same cycle as for GRβ. The reassociation temperatures for M-GR and U-GR were 54°C and 52°C, respectively.

### Flow cytometry

Annexin V-FITC/PI apoptosis detection kit (BD Biosciences Clontech, Palo Alto, CA, USA) was utilized for flow cytometry to determine the percentage of apoptotic cells. Cells were harvested and stained following the manufacturer's protocol [24]. In brief, 1×10^5^ cells were incubated with 5 μL of Annexin V-FITC and 10 μL 20 μg/mL PI for 15 minutes at room temperature in the dark. Each sample was added with 500 μL of PBS binding buffer and analyzed using flow cytometry (BD Biosciences Clontech) within 30 minutes.

### DNA fragmentation

LoVo cells were incubated with or without 1×10^−4^ M dexamethasone (Sigma-Aldrich) in media without FBS for 24, 48, 72 hours. Cells were collected and lysed by DNA lysis buffer (10 mM Tris-HCl, pH 7.5, 10 mM EDTA, pH 8.0, 0.5% Triton X-100, 20% SDS, 10 mg/mL proteinase K). DNA was extracted using phenol-chloroform method. Then, separation by electrophoresis was performed on 2% agarose containing ethidium bromide [23, 25].

### Hoechst staining

LoVo cells were incubated with or without 1×10^−4^ M dexamethasone (Sigma-Aldrich) in media without FBS for 72 hours. Then, cells were incubated with 1 μg/mL Hoechst 33342 (Sigma-Aldrich) at 37°C for 15 minutes and observed under an inverted fluorescence microscope immediately [13].

### TUNEL

LoVo cells were incubated with 1×10^−4^ M dexamethasone (Sigma-Aldrich) in media without FBS for 72 hours. Apoptotic cells were detected using the terminal deoxynucleotidyl transferase-mediated nick end-labeling (TUNEL) stain using an *In Situ* Cell Death Detection Kit, PO (Roche, Germany) following the manufacturer's protocol, as described previously [14].

### MTT assay

Growth inhibition and drug sensitivity were assayed using the MTT assay as previously described [21, 26]. In brief, cells were incubated with MTT (BD Biosciences Clontech) at 37°C for 4 hours and formazan crystals were dissolved using DMSO (Sigma-Aldrich). The absorbance was measured at 490 nm using a microplate reader. The absorbance was normalized to their control cells, respectively.

### Western blot

Cells were lysed in RIPA buffer with protease inhibitors and phosphatase inhibitors [15–16]. The protein was resolved by SDS/PAGE and blotted on PVDF membranes (Millipore, Bedford, MA, USA)[15–16]. The PVDF membranes were incubated with specific primary antibodies at 4°C overnight [15–16]. After incubation with HRP-linked secondary antibodies, immunoreactive proteins were visualized using DAB (Beijing Zhongshan Jinqiao biotechnology Co., Ltd.).

Primary antibody against GRα was from Santa Cruz Biotechnology. IκBα, phospho-p65 and cleaved caspase 3 were from Cell Signaling Technology (Beverly, MA, USA). NF-κB p65, Bcl-2, β-actin and HRP-linked secondary antibodies were from Beijing Zhongshan Jinqiao biotechnology Co., Ltd.

### EMSA

Nuclear extracts were prepared following the protocol of Nuclear Extraction kit (Keygen Biotech, China). EMSA was performed according to the manual of EMSA kit (Keygen Biotech, China). In brief, 2 μg nuclear extracts were incubated with ^32^P-labeled double-stranded NF-κB probe (5'-AGCTTAGAGGGGACTTTCCGAGAGGA-3’; 5'-TCCTCTCGGAAAGTCCCCTCTAAGCT-3’. The underlined indicates NF-κB binding site.) for 20 minutes at 37°C. DNA/nuclear protein complexes were resolved in a native 5% acrylamide gel and the gel was subjected to autoradiography [17].

### Knockdown of genes

GRα was knocked down by plasmid mediated shRNA interference using pGenesil-1 (Genesil Biotechnology Co, Ltd., China) according the manual [27]. The GRα-targeting vector or an empty vector (control) was transfected into LoVo cells using Lipofectamine^®^ 2000 (Invitrogen) according to the manufacturer's instructions [14, 21]. The sense sequence of the shRNA targeting GRα is GAGATCATATAGACAATCA.

GRβ was knocked down by the siRNA sequences. The sense sequence is 5’-GGCUUUUCAUUAAAUGGG ATT-3’ and the anti-sense sequence is 5’-UCCCAU UUAAUGAAAAGCCTC-3’. The sense sequence of scrambled RNAs as a control is 5’-UUCUCCGAA CGUGUCACGUTT-3’ and the anti-sense sequence is 5’-ACGUGACACGUUCGGAGAATT-3’. All siRNA duplexes were chemically synthesized by the Shanghai GenePharma Company (China).

NF-κB p65 was knocked down by retroviral mediated shRNA interference as previously described [14]. The sense sequence of the shRNA targeting p65 is GCCCTATCCCTTTACGTCA.

### Microarray dataset of selection and analysis

Microarray dataset of selection and analysis were performed as previously described [28]. The microarray datasets of CRCs patients and CRC cell lines microarray analyzed here were obtained from the publicly available NCBI Gene Expression Omnibus (GEO, http://www.ncbi.nlm.nih.gov/geo/) databank. Accession numbers were GSE39582, GSE36133 and GSE17536.

These datasets were selected according to the following criteria: (1) Microarray was analyzed using Affymetrix Human Genome U133 Plus 2.0 Array. (2) Raw data are available. (3) Both the control and the standard were used. Preprocessing of raw data and quality control was accomplished using robust multichip average (RMA) as previously described [29–30]. The heatmap was performed using the pheatmap package in R as previously described [31–32]. Apoptosis genes and cell cycle signature genes were obtained from HALLMARK APOPTOSIS and KEGG CELL CYCLE Gene sets in Molecular Signatures Database(v5.1) (Broad Insititute)[33].

### Statistical analyses

The data shown represent the mean or the mean ± standard error. Statistical differences were analyzed by Student's t-test, one-way ANOVA, Kruskal-Walis test or Mann-Whitney test. *p*< 0.05 was considered statistically significant.

## SUPPLEMENTARY MATERIALS FIGURES



## Abbreviations

GR: glucocorticoid receptor
H&E: hematoxylin and eosin
M: mol/L
5-Aza: 5-Aza-2’-deoxycytidin
RT-PCR: Semi-quantitative reverse transcription polymerase chain reaction
MSP-PCR: Methylation-specific PCR
PI: propidium iodide
EMSA: Electrophoretic mobility shift assay
sh: small hairpin
siRNA: small interfering RNA
IC_50_: half maximal inhibitory concentration
NF-κB: nuclear factor κ B
IκB: Inhibitor of κB
