# Supplementary material for: Dexamethasone affects cell growth/apoptosis/chemosensitivity of colon cancer via glucocorticoid receptor α/NF-κB
Source: Oncotarget. 2017 Jun 28;8(40):67670–83. doi: 10.18632/oncotarget.18802 (PMC5620202; doi:10.18632/oncotarget.18802)
Supplement: Supplementary file 1 [file oncotarget-08-67670-s001.pdf]

www.impactjournals.com/oncotarget/ Oncotarget, Supplementary Materials 2017

Dexamethasone affects cell growth/apoptosis/chemosensitivity of colon cancer via glucocorticoid receptor α/NF-κB

SUPPLEMENTARY MATERIALS

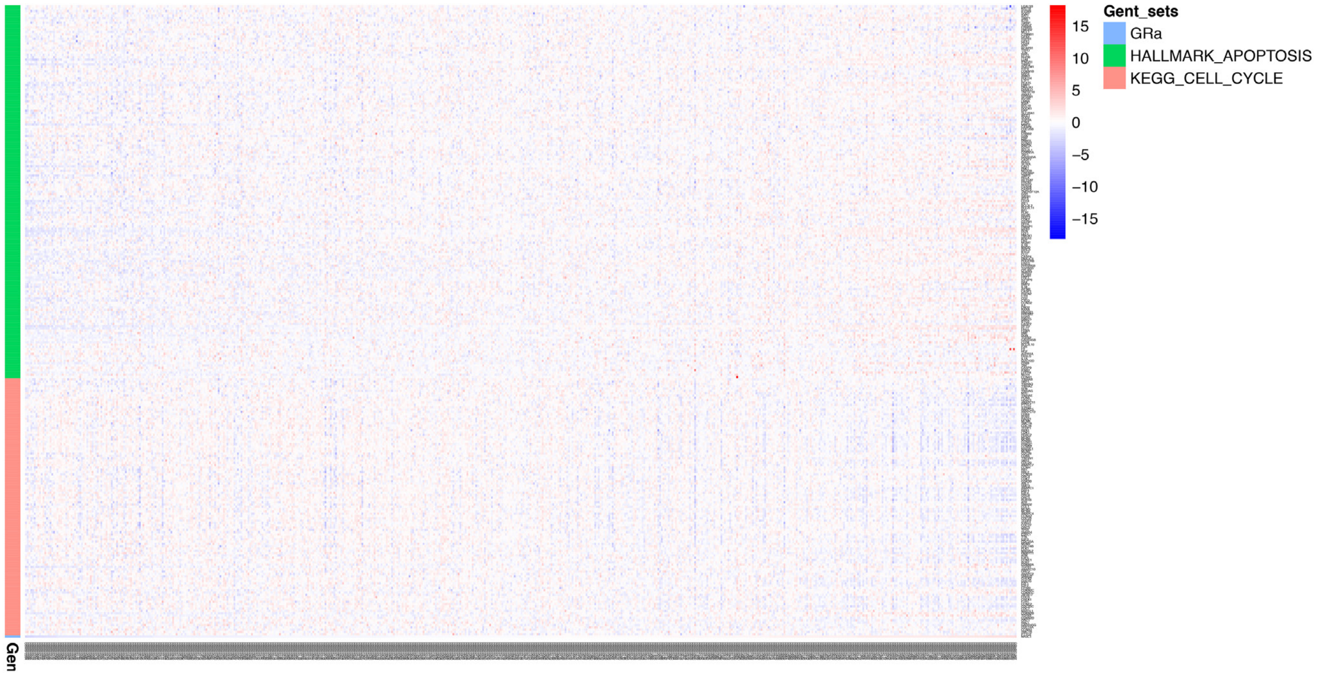

Supplementary Figure 1: The heatmap showed the correlation between GRα and apoptosis genes, and cell cycle genes.

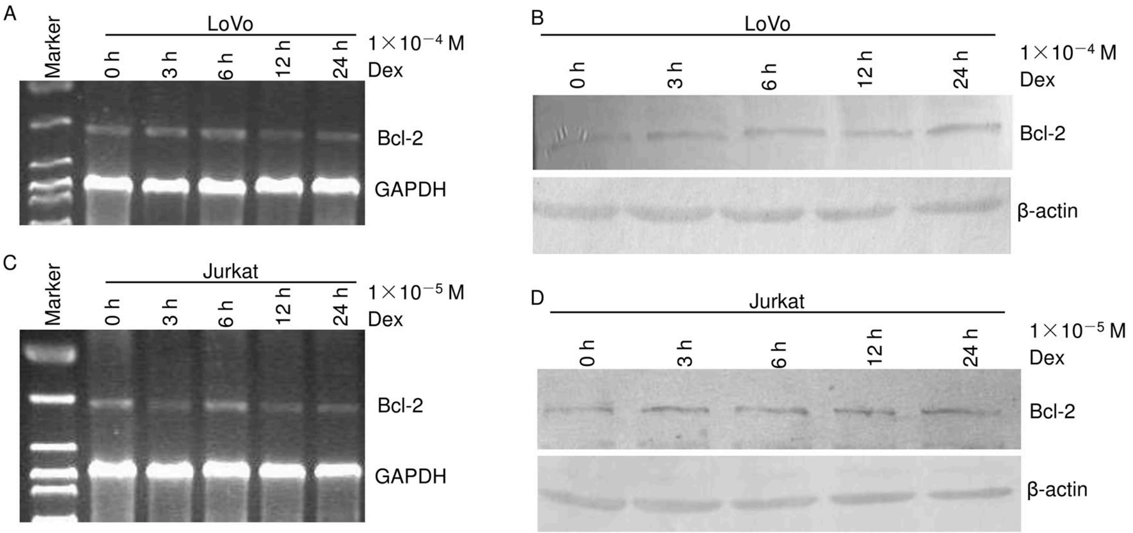

E

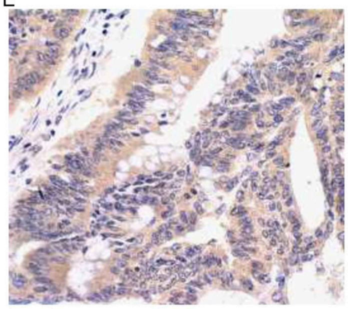

F

		GR		*P*
		Positive n(%)	Negative n(%)	
Bcl-2	Positive, n=50	28(56.0)	22(44.0)	0.643
	Negative, n=11	7(63.6)	4(36.4)	

Supplementary Figure 2: The role of bcl-2. (A) RT-PCR. **(B)** Western blot. **(C)** RT-PCR. **(D)** Western blot. **(E)** bcl-2 immunohistochemistry of colon cancer. **(F)** Correlation between GRα and bcl-2 in colon cancer were analyzed using immunohistochemistry.
